# The Pandemic Century: One Hundred Years of Panic, Hysteria and Hubris

**DOI:** 10.3201/eid2606.191739

**Published:** 2020-06

**Authors:** Nkuchia M. M’ikanatha

**Affiliations:** Pennsylvania Department of Health, Harrisburg, Pennsylvania, USA

**Keywords:** pandemic preparedness, respiratory diseases, influenza, Legionella, SARS, viruses, bacteria, zoonoses

Long before microbes were discovered, a sudden occurrence of disease in a society would trigger fear, accompanied by rational and irrational responses. A mysterious, highly contagious disease that spread across the Mediterranean and killed a quarter of the population of Athens is the first recorded pandemic (*1*).

*The Pandemic Century: One Hundred Years of Panic, Hysteria and Hubris* by Mark Honigsbaum, a medical historian at the City University of London (UK), focuses on large-scale outbreaks since 1900. The author uses 9 examples to shine a light on epidemiological blind spots to avoid in future investigations. For example, he suggests that the initial lackluster response to the 1918 influenza pandemic can be attributed in part to false confidence in Richard Pfeiffer’s 1892 discovery of *Bacillus influenzae*, now known as *Haemophilus influenzae.* We later learned that *H. influenzae* occasionally is found in persons with influenza but was not the cause of the pandemic.

Through colorful and engaging narrative, Honigsbaum probes the social context of early 20th Century America that led investigators to attribute differences in pneumonia rates among African Americans to racial factors compared with white soldiers at Camp Funston, Kansas, thought to be an early site of the 1918 influenza pandemic. He details how public hysteria, fear, and conspiracy theories hindered public health responses in other outbreaks, such as in the 1976 Legionnaires’ disease outbreak in Philadelphia, Pennsylvania. He includes laboratory and epidemiological investigations that led to identification of *Legionella pneumophila* in 1977 (Figure). The film, Influenza 1918, which aired on the Public Broadcasting Service, also profiles the pandemic (*2*).

**Figure:**
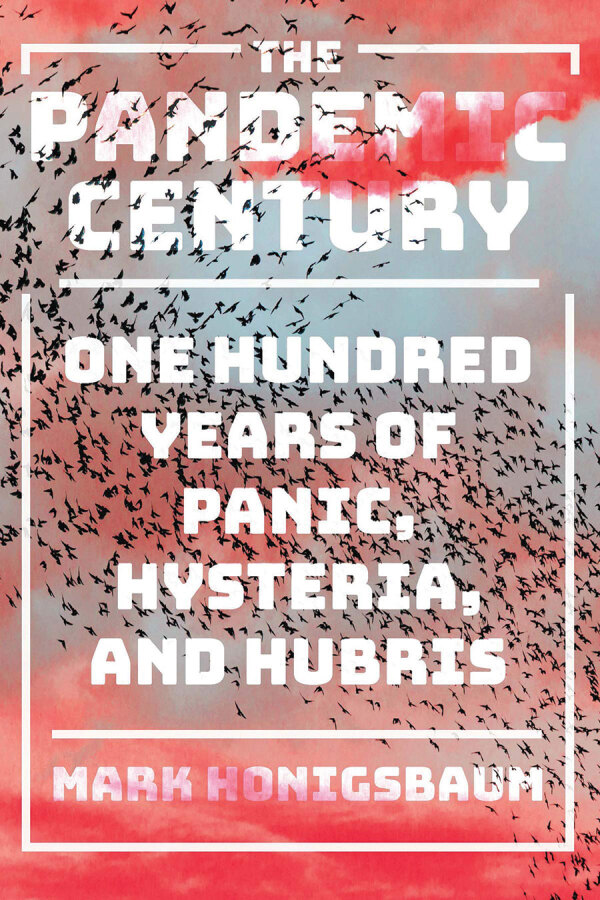
The Pandemic Century: One Hundred Years of Panic, Hysteria and Hubris

The book at times offers a gloomy assessment of how, in today’s era of international travel, ecological disturbances and human behaviors have tipped the scales in favor of pathogens. It also highlights lapses that occurred during outbreak investigations, such as how mishandled laboratory samples delayed recognition of severe acute respiratory syndrome (SARS) in 2002 and how cross-border travel and the lack of appreciation for local cultural practices by investigators facilitated spread of Ebola virus in West Africa during 2014–2016.

The text concludes with a description of global efforts to anticipate pandemics, such as Resolve, a public health initiative supported by philanthropic institutions including the Bill and Melinda Gates Foundation. Honigsbaum notes uncertainties and gaps here too, such as Madagascar’s ineligibility to receive support to respond to a massive *Yersinia pestis* outbreak from a World Bank fund. The fund was established after the Ebola outbreak in West Africa and only can be used for diseases caused by viruses that have certain characteristics and threaten to spread internationally (*3*).

This book appealed to me because it condenses events spanning a century into readable segments told by an author known for in depth historical accounts. *The Pandemic Century* could appeal to diverse categories of readers, including epidemiologists, public health workers, students, and anyone interested in understanding why, despite impressive gains in global public health preparedness and advances in disease prevention and control, pandemics continue to surprise and terrify us.

## References

[1] Littman RJ. The plague of Athens: epidemiology and paleopathology. Mt Sinai J Med. 2009;76:456–67. 10.1002/msj.20137 19787658

[2] Influenza 1918. American Experience. Public Broadcasting Service. 2010 Jan 18 [cited 2020 Apr 14]. https://www.pbs.org/video/american-experience-influenza-1918

[3] The World Bank. World Bank launches first-ever pandemic bonds to support $500 million pandemic emergency financing facility [cited 2019 Nov 30]. https://www.worldbank.org/en/news/press-release/2017/06/28/world-bank-launches-first-ever-pandemic-bonds-to-support-500-million-pandemic-emergency-financing-facility

